# The VUCA world and paradoxical dilemma on predicaments of foreign aid withdrawal or adjustment: problematisations, implications, and lessons

**DOI:** 10.7189/jogh.15.03035

**Published:** 2025-08-15

**Authors:** Laston Gonah, Londele Tyeshani

**Affiliations:** Department of Public Health, Walter Sisulu University, Mthatha, South Africa

## Abstract

The already impending volatile, unpredictable, complex, and ambiguous (VUCA) world, driven in part by shifts and uncertainty in global, political, and financial markets, is becoming increasingly apparent, thereby rationalising the importance of vigilance, preparedness, and resilience by aid stakeholders. The implications this has for global health and security, governance, and development deserve critical consideration in efforts to protect public well-being and achieve or sustain resilience. The recent turn of events regarding the United States Agency for International Development’s humanitarian aid withdrawal/adjustment notice can be seen as an omen of an uncertain future if the implications are disregarded. Establishment of foreign aid programmes is motivated by several defining principles and factors that must be considered when critiquing problematisations related to their eventual adjustment/withdrawal. We designed and proposed a framework for critiquing motives, responsibility, benefits, opportunities, lessons, and dos (MR-BOLD), serving as a diagnosis and response planning tool to address lingering questions about the VUCA world-induced effects, such as aid adjustments/withdrawals. We should nudge and spread world consciousness that the impending VUCA world demands an antidote in the form of critical thinking, religious observance of corporate governance principles – transparency, accountability, fairness and responsibility, and measures to ensure efficiency in how all natures of relationships are managed to optimise their predictive success, lest our failures besiege us. Development stakeholders should span boundaries to critical thinking and innovation, such as diverse knowledge, approaches, perspectives, and culture, to consider the best ways to integrate, organise, and deploy available resources and opportunities in the quest for resilience.

The establishment of foreign aid projects is motivated by various factors and reasons best known by the funding organisations. For instance, the United States Agency for International Development (USAID) humanitarian aid originated from the USA’s Foreign Assistance Act of 1961 as part of the USA government’s official foreign policy implementation strategy, which has grown to be the largest aid worldwide with an annual budget of over USD 47 billion [1–3]. Over the years since its inception, USAID has changed in size, budget, and focus. In more recent years, USAID humanitarian aid has primarily focussed on promoting development and capacity building to over 100 countries that are strategically important to the USA government and countries in conflict, establishing and strengthening a network of partnerships with non-governmental organisations and private enterprises in the process [3]. Most of USAID’s financial aid beneficiaries are in Africa, Latin America, the Middle East, Asia, and Eastern Europe [3–5]. The recent turn of events, in the form of an unprecedented and abrupt notice for withdrawal of USAID humanitarian aid announced in January 2025, attracted mixed reactions across the world [2,4] – a shock and perceivably an inhumane act to some, while providing an opportunity for critical thinking and learning for others. We did not aim to directly address or respond to the USAID humanitarian aid adjustment/withdrawal notice; instead, we tapped into this recent predicament to discuss and prepare for similar scenarios.

Critical thinking and planning are crucial for understanding and responding to predicaments, such as the notice for USAID humanitarian aid adjustment/withdrawal. We present and discuss personal opinions – at the time of press, on this and other similar predicaments, critically considering the associated problematisations and implications, outlining lessons that can be drawn from this and similar occasions. The impact of foreign aid withdrawal on global health and security, governance, and development deserves critical consideration in efforts to protect public well-being and achieve or sustain resilience in the face of the already impending volatile, unpredictable, complex, and ambiguous (VUCA) world, characterised by economic recessions and austerity measures [6,7].

Given the predicaments of foreign aid withdrawal, we propose a framework for critiquing the motives, responsibility, benefits, opportunities, lessons, and dos (MR-BOLD), as an objective diagnosis and response planning tool to VUCA world-induced effects such as aid adjustments/withdrawals. We believe that critiques must be balanced and devoid of emotional kidnappings or personal interests. They should not be biased towards any financial, individual, or political motives, but rather factual/technical and practical ones.

Critiquing aid-related predicaments in a VUCA world requires an understanding of the:

1. **M**otivation behind the establishment and eventual adjustment/withdrawal of the aid;
2. **R**esponsibility, *i.e. *who is attributable as responsible for the adjustment/withdrawal;
3. **B**enefits, *i.e. *who benefits from the aid, or is affected by its adjustment/withdrawal;
4. **O**pportunities that are presented by the aid to the aid stakeholders, or that can be explored to mitigate or address the predicament;
5. **L**essons drawn from the predicament, all interacting to guide/inform on what to **D**O in responding to the predicament.

We have coined this MR-BOLD process (**Figure 1**), and in this discussion, we will not go into detail in answering the questions contained in the MR-BOLD framework, as our focus is not on targeting a specific occasion but instead on proposing it to guide the understanding and management of aid adjustments/withdrawals in a VUCA world.

**Figure 1:**
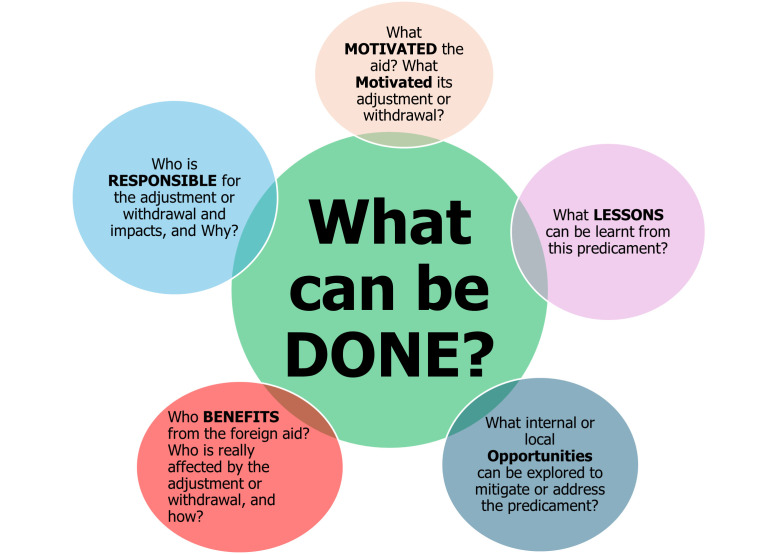
MR-BOLD framework for the diagnosis and management of aid adjustments/withdrawal predicaments in a VUCA world. MR-BOLD – motives, responsibility, benefits, opportunities, lessons, and dos. VUCA – volatile, unpredictable, complex, and ambiguous

## Understanding foreign aid adjustments/withdrawals using MR-BOLD

### What motivated the aid creation, and its eventual adjustment or withdrawal?

It is pertinent to understand the contextual history of a given aid, that is, the principles and conditions underpinning its formation and existence. Most foreign aid projects have been constantly changing over time, as the capacity, strategic interests, and priorities of the funder shift or change in response to circumstantial alterations [1,6,7]. Some foreign aid agencies have gained complete autonomy from funding governments, while others have not, thereby differentially affecting how they are perceived and understood by their governments and stakeholders, which may affect the decision to adjust/withdraw aid. There are also corporate governance and management issues to consider, specifically how efficiently the organisation implementing the aid is run. Most foreign aid programmes are built on corporate governance principles of transparency, fairness, accountability, and responsibility, which must be monitored and evaluated over time. Hence, understanding the motives, principles, and conditions underpinning the formation and existence of an aid is essential in understanding why it may have been adjusted/withdrawn. Foreign aid adjustment/withdrawal is inevitable, especially when any key attribute underpinning its existence is no longer consistent with the sole funder’s original intentions.

### Who is responsible for the adjustment/withdrawal and the resulting impacts, and why so?

Foreign aid adjustments/withdrawals are usually accompanied by a disagreement from different stakeholders, naturally due to lost stake. The foreign aid value chain is composed of various types of stakeholders. These may include the funding government and its citizens, or the funding organisation, the beneficiary government/community, and its citizens, the targeted/intended beneficiaries, and the workforce/human resource implementing the projects. These stakeholders may have shared or different interests, roles, decision-influencing powers, and views/perceptions. Still, they all have some responsibility and accountability over the life of a foreign aid project, though maybe different in scope and scale. It implies that any or all the stakeholders can be responsible for a foreign aid’s withdrawal/adjustment. Above all, it is essential to consider the circumstances behind or how the adjustment/withdrawal would have been done – either abruptly or gradually, with or without notice/consensus. Given the involvement of several stakeholders, all the stakeholders should be informed, with the maximum possible time allowance, of any emerging developments/decisions that may affect their stake or future in the foreign aid value chain to better prepare/adjust to the changes. Therefore, answering the question on who is responsible for aid adjustment/withdrawal also requires a complete understanding of the foreign aid value chain, in terms of its motive, purpose, structure, composition, and function, as well as the prevailing internal and external operating environment or circumstances.

### Who benefits from the foreign aid? Who is affected by the adjustment/withdrawal and how?

Foreign aid projects are based on specific interests and certain agreed-upon or conditional principles among the stakeholders involved. The stake that each stakeholder perceives in the foreign aid establishment may reflect their perceived benefits or value, which can vary over time and are not necessarily recognised or valued equally by all stakeholders. Hence, the interaction of the foreign aid value chain components with the internal and external circumstances over time can shape what each stakeholder can perceive as a benefit that may influence their perception of its value.

The stakeholder’s influence and perceived or actual benefits often shape the course of aid, with funders – typically governments and their citizens– holding greater control than other stakeholders who have less influence despite being in greater need. It is also important to note that the need is not always fairly problematised and benefits correctly represented or clearly defined, understood, and measurable by all the stakeholders involved. What the problem is described to be – problem problematisation, is not always a true reflection of the actual problem [8]. There is a postulation that foreign aid projects can be weaponised to expand global political influence and dominance at the expense of humanitarian principles, ethics, and human rights [9–11]. This may sometimes result in foreign aid problematisations that are skewed to hide the hidden intentions of the funder – such as expansion of global political influence and dominance, but to headline the apparent need of the aid recipient – such as developmental/relief aid.

What counts as an aid benefit or value, or who counts as a victim of aid adjustment/withdrawal, might be subjective and partly dependent on the extent to which corporate governance principles of transparency, accountability, fairness, and responsibility are upheld in the aid value chain. All things being equal, it would make sense to say that most stakeholders are affected by aid adjustment/withdrawal when it occurs. The question of who suffers or is affected when aid is adjusted/withdrawn can be complex, due to the inherent presence of different stakeholders with varying interests that are not always clearly known. Answering this question requires answers from other questions, such as ‘How does aid flow along its value chain, how is it allocated, and how is it accounted for?’, ‘Who are the intended beneficiaries, how much of the aid filters to the intended beneficiaries, and how do the beneficiaries feel about the aid in terms of perceived benefits and value?’, ‘Is how the aid is administered consistent with the funders’ original intentions?’, and ‘What motivated the adjustment or withdrawal?’.

Value chains are often infested with deceit and disguise, malpractices, dominations, and grandstanding, where the troubles of the poor or vulnerable stakeholders may be used as an opportunity to advance the selfish desires of the stronger ones [2,10,12]. Consciousness to the plight of the poor and allegedly pro-poor problematisations of needs and benefits is an essential resource in critical considerations related to aid adjustments/withdrawals. Those who benefit from the aid or *status quo* may manipulate beneficiary approval or dissent to justify its continuation and deceitfully enhance their gains. We believe that, if there can be disguised motives, there can also be disguised victims. If aid problematisations can be skewed to conceal the real motives, actual victims of aid withdrawal can also be hidden. The solution to these and other concerns lies in the implementation and observance of corporate governance principles of transparency, accountability, fairness, and responsibility, without which, efficient operation will be challenging to realise.

### What internal or local opportunities can be explored to mitigate or address the predicament?

Foreign aid can be considered an opportunity to enhance resilience, providing a chance to explore ways to achieve it. Opportunities to respond to aid adjustments/withdrawals – whether abrupt or gradual – should be analysed and identified as a necessary part of any effective resilience strategy. The desirable scenario in this VUCA world is when aid beneficiaries are more preoccupied with resilience and strategies to achieve it, rather than continuously feeling more vulnerable and entitled to receive aid. In the face of the increasingly VUCA global landscape, a paradigm of entire dependency on humanitarian aid must shift towards achieving resilience and, where possible, considering adaptive response strategies [13]. This can include having aid withdrawal moderating opportunities or systems in place, such as budget or economic reforms, strengthening local funding or local private sector engagement, and regional capacity building or economic empowerment. Foreign aid arrangements must include a stakeholder-co-constructed resilience plan to prevent desperation when aid is halted for any reason. The relevant questions are: ‘When aid was there, did it co-exist with a resilience strategy and why?’ and ‘When aid was withdrawn, how much progress had been made towards achieving resilience and how much support is required to complement the existing initiatives or opportunities?’. The opportunities available to address a predicament of aid adjustment/withdrawal may originate from reorientation of priorities and reorganising locally available resources and innovations to achieve resilience, as an essential component of a resilience strategy. We note that there are possible exceptional scenarios of total to near-total economic, political, and social shutdown that may persist over extended periods, rendering resilience almost impossible and foreign aid the only hope. Despite the precarious situation, foreign aid is inherently volatile and transient, rendering resilience strategic planning indispensable [1].

### What lessons can be learnt from this predicament?

When aid is adjusted/withdrawn, each stakeholder must take responsibility for responding and adapting to the change that all stakeholders should have anticipated and prepared for, considering the prevailing VUCA world. By its nature, unless otherwise specified, foreign aid is transient and condition-bound, often designed to provide time-bound relief to the aid recipient, giving them room to leverage the assistance in building/strengthening their capacity and resilience. Therefore, foreign aid stakeholders must not consider the aid as permanent/continuous, reliable, and an entitlement, thereby becoming overly dependent on it. Stakeholders need to note that the capacity, motives, interests, or priorities of funders can change at any time due to foreseeable or unforeseeable circumstances, alterations, or influences.

Any foreign aid recipient needs to have a resilience strategy, detailing how they plan on achieving autonomy and resilience over time, even in the event of abrupt aid adjustments/withdrawals. Foreign aid programmes can be considered a valuable asset to enhance the host’s resilience strategy, where progress towards resilience goals must be tracked and accounted for over time. A resilience strategy must deliberately and critically assess a society’s or community’s strengths, weaknesses, opportunities, and threats, and be guided by a clear vision, mission, goals, objectives, and action plan [14].

### What can be done?

Responding to aid adjustments/withdrawals in the VUCA world requires world consciousness, critical thinking, and consideration of all the issues discussed. Insights generated from critiquing motives, responsibilities, benefits, opportunities, and lessons associated with aid can serve as valuable capital for informing well-reasoned conclusions and distilled ideas on what can be done as a response to aid adjustments/withdrawals.

Governments, aid, and community/societal stakeholders must transcend boundaries to critical thinking and innovation, such as employing diverse knowledge, approaches, expertise, perspectives, or cultures, and considering the best ways to integrate, organise, and deploy available resources and opportunities in their quest for resilience. This would require deliberate efforts to span boundaries and utilise all the existing approaches, principles, tools, and resources, integrating and organising them to achieve resilience (Figure S1 in the **Online Supplementary Document**).

We believe that achieving resilience requires consideration of several ideas and deciding how they can be best organised and deployed for effectiveness:

1. Exploration and utilisation of relevant and existing tried and tested principles, strategies, and actions that are informed by theory and practice, such as:

- Radical community developmental principles: strong commitment to collective action for justice, promoting critical consciousness and participation in local issues to encourage empowerment, critical analysis of societal power and discrimination, clear understanding of underlying ideas that cause/promote issues and predicaments, and action targeting root causes of the problem rather than symptoms [15].
- Adaptive response strategies: putting in place aid withdrawal moderating systems, such as budget or economic reforms, strengthening local funding or local private sector engagement, and regional capacity building or economic empowerment [13].
- Equity promotion principles or action areas: building a healthy public policy, creating supportive environments; reorienting health services, developing personal skills and strengthening community action, based on principles of community participation, partnership, and empowerment and equity through three basic strategies – to enable, advocate, and mediate [16,17].

2. Prioritising research and development: advocacy, policy and planning, intervention, monitoring and evaluation, through efficient utilisation of available resources, innovations, and partnerships towards achieving and sustaining community or societal resilience.

3. Making practical steps to ensure political and corporate governance, political commitment, and transparency towards development and resilience

## CONCLUSIONS

The time has come to nudge and spread world consciousness that the impending VUCA world demands an antidote in the form of critical thinking, religious observance of corporate governance principles – transparency, accountability, fairness, and responsibility, and measures to ensure efficiency in how all natures of relationships are done to optimise their predictive success, lest our failures besiege us. Development stakeholders should span boundaries to critical thinking and innovation, such as diverse knowledge, approaches, perspectives, and cultures, to consider the best ways to integrate, organise, and deploy available resources and opportunities in the quest for resilience.

## Additional material


Online Supplementary Document
