# Supplementary material for: The VUCA world and paradoxical dilemma on predicaments of foreign aid withdrawal or adjustment: problematisations, implications, and lessons
Source: J Glob Health. 2025 Aug 15;15:03035. doi: 10.7189/jogh.15.03035 (PMC12355440; doi:10.7189/jogh.15.03035)
Supplement: Online Supplementary Document [file jogh-15-03035-s001.pdf]

Supplement to: Gonah L, Tyeshani L. The VUCA world and paradoxical dilemma on predicaments of foreign aid withdrawal/adjustment: problematisations, implications, and lessons. J Glob Health. 2025;15:03035.

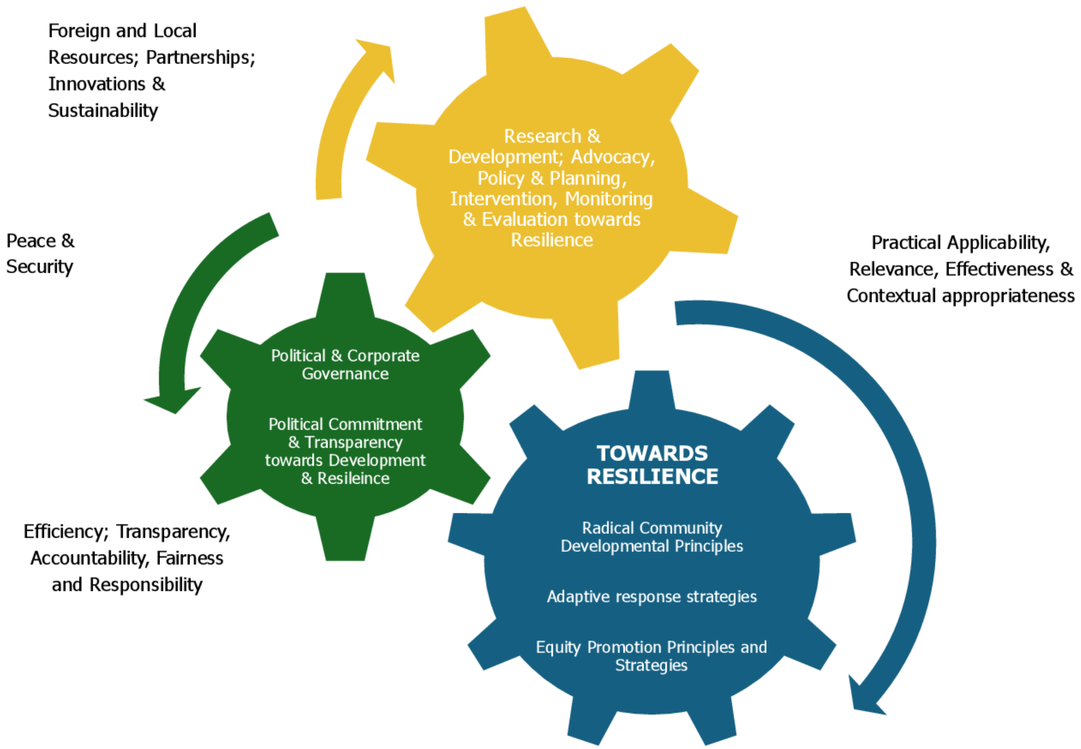

Figure S1. Approaches towards resilience.
